# Full recovery of hip muscle strength is not achieved at return to sports in patients with femoroacetabular impingement surgery

**DOI:** 10.1007/s00167-018-5337-0

**Published:** 2018-12-12

**Authors:** Sam Hallberg, Mikael Sansone, Jesper Augustsson

**Affiliations:** 1 Department of Sport Science at the Faculty of Social Sciences, Linnaeus University, 391 82 Kalmar, Sweden; 2 Department of Orthopaedics, Institute of Clinical Sciences, Sahlgrenska Academy, Gothenburg University, Sahlgrenska University Hospital (Mölndal), Mölndal, Sweden

**Keywords:** Femoroacetabular impingement, Hip strength, Sports, Rehabilitation

## Abstract

**Purpose:**

The purpose of this study was to study dynamic hip  external rotation strength in patients with Femoroacetabular impingement surgery (FAI) syndrome who have undergone unilateral arthroscopic treatment and returned to sports.

**Methods:**

A cross-sectional study was performed using an observational group (*n* = 22) and a matched control group (*n* = 22). Dynamic external rotation strength of the hip was measured using the Augustsson Strength Test, which has shown high reliability for examining side-to-side differences in hip muscle strength.

**Results:**

Dynamic hip external rotation strength was significantly lower in the arthroscopically treated hip compared with the non-treated hip within the observational group (*p* < 0.004).

**Conclusion:**

This cross-sectional study shows that at return to sports, patients who have undergone unilateral arthroscopic treatment for FAI syndrome do not have adequate hip muscle strength recovery. Rehabilitation protocols should, therefore, emphasise post-operative strength training of the hip muscles. Additional research is needed to determine the consequences of reduced hip strength for the long-term outcome after arthroscopically treated FAI. Clinical relevance: The results of this study underline the importance of post-operative strength training prior to returning to sports in patients with femoroacetabular impingement surgery.

**Level of evidence:**

III.

## Introduction

Femoroacetabular impingement (FAI) is an anatomical hip deformity, which causes a conflict between the femur and the acetabulum [24]. FAI is a syndrome divided into CAM-type, Pincer-type and mixed-type. CAM involves a deformity of the femoral neck while Pincer refers to a deformity of the acetabulum [6, 7, 25, 36]. FAI syndrome could result in damage to the labrum and joint cartilage, hip pain, reduced mobility, muscle strength deficit of the hip, altered movement patterns and reduced hip functions [3, 6–8, 11, 16, 17, 19, 24, 36]. FAI syndrome is a strong risk factor to develop hip osteoarthritis [6, 16, 25]. The exact prevalence of FAI syndrome has not been thoroughly determined [24, 31]. FAI syndrome is common in young adults who are healthy, active and participate in sports [32], although the exact prevalence of FAI syndrome has not been determined [23, 24, 31].

Surgical treatment of FAI syndrome using arthroscopy has become increasingly common even though conservative treatment is often recommended prior to surgery [12, 13]. There are studies in the literature regarding the long-term effects of arthroscopic treatment of FAI syndrome [25, 29]. In these studies, self-assessment forms were used during follow-ups, whereas strength tests were not performed. The outcome of arthroscopy seems to be generally good [4, 32] with a gradual return to sports at 4–6 months after surgery [6]. Because studies in which hip strength testing is performed following FAI surgery are lacking in the literature, it is not clear whether these patients are ready to return to sports.

Sufficient hip muscle strength plays an important role for the athlete from a performance and injury prevention perspective [5, 10, 16, 20, 21, 33]. Reduced hip strength can be associated with an increased risk of various conditions of the lower extremities, for example iliotibial band syndrome [1, 26], anterior cruciate ligament (ACL) tear [15] and patellar tendinopathy [37].

Evidence-based guidelines for rehabilitation after arthroscopic treatment for FAI syndrome are currently lacking in the literature [4, 9, 11, 14], as well as return-to-play protocols for athletes [30]. Very few studies have been published regarding physiotherapy protocols after arthroscopic treatment of FAI syndrome [11].

Concerning both operated and non-operated individuals with FAI syndrome, studies have shown reduced strength and functionality of the hip evaluated using self-assessment forms [4, 13], isometric tests [8, 13, 24, 28] and functional tests [17]. However, none of these studies have tested dynamic hip muscle strength at return to sports after arthroscopic FAI syndrome treatment, compared to the uninvolved hip or versus normal controls. Therefore, questions of clinical relevance such as the effect of strength training during FAI rehabilitation and whether patients display normal hip muscle strength symmetry at return to sports are largely unanswered. The primary aim of this study was to measure dynamic hip muscle strength at return to sports in patients with FAI syndrome who have undergone unilateral arthroscopic treatment. A secondary aim of this study was to compare the patients’ hip muscle strength with a matched control group.

## Materials and methods

**Fig. 1:**
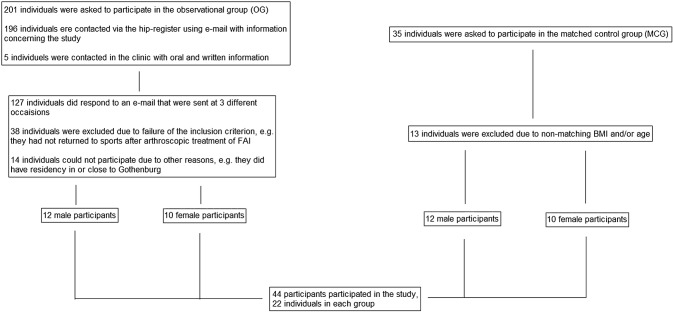
Flow chart of the recruitment process

In this cross-sectional study patients were divided into an observational group recruited from a local hip arthroscopy registry (Ortho Center IFK Clinic, Gothenburg, Sweden) and a matched control group, recruited through local physiotherapists and sports clubs. The patients in the observational group were contacted via e-mail with information regarding the study. A total of 196 e-mails were sent to 135 men and 61 women. The participants in the matched control group were asked in person by the physiotherapist in the clinic. The complete recruitment process is presented in Fig. 1.

Adult men and women who had undergone unilateral arthroscopic treatment of FAI syndrome and returned to their previous sports at a minimum of 4 months postoperatively were included in the study. Patients with bilateral arthroscopic treatment of FAI syndrome at the same time or at a different time, and on-going treatment of other health conditions were excluded. The matched control group consisted of 22 individuals with healthy, untreated hips, all active in sports. They were matched regarding Body Mass Index (BMI) and age. BMI was set to maximum 5% above or below the relevant individual in the observational group. The age limit was set to max 5 years older or younger than the relevant individual in the observational group.

### Test procedures

The hip muscle strength test, Augustsson Strength Test, used in the study was recently developed and has shown high reliability (intra-class correlation coefficients ranged 0.93–0.94) for measuring unilateral hip strength, and thus assess differences between sides [2]. It is a clinical muscle function test for assessment of dynamic hip external rotation strength. In this test, a device is used that combines a strap connected in series with an elastic resistance band loop, and a measuring tape connected in parallel with the elastic resistance band. The test was carried out with the patient side lying, positioned in 45° of hip flexion and the knees flexed to 90° with the device firmly fastened proximally across the knees. The subject then exerted maximal concentric hip external rotation force by pressing the upper leg against the device while keeping the feet together, thereby extending the elastic resistance band. The displacement achieved by the subject was documented by the tape measure in cm. The Augustsson Strength Test was performed on both hips (one at a time) on each patient.

To ensure good form and proper test technique, the patient was not allowed to rotate the body or use the arms to help in any way during the test. The patient performed the test three times per side with 1 min of rest between each time, and the best trial for each side was used for data analysis. Which side was tested first was randomly selected using a computer program with a random number generator (Excel, Microsoft, USA).

The instrument used for the Augustsson Strength Test consisted of a common non-elastic strap (Arno, Sweden), an elastic rubber band made for training/rehab (model Refit Rubberband, Sweden) and a measuring tape (Profit, Germany). A green rubber band (medium) was used for 43 participants, for one patient in the observational group, however, the medium band did not offer sufficient resistance and a blue band (heavy) was used instead. The corresponding force production was calculated according to a previously described method by Augustsson [2] in which elastic resistance band displacement was converted to force. Using this method, it was possible to return results in kg for any given value in cm. All the tests, calibrations and conversions were conducted by the same test leader.

Aside from the hip muscle strength test, the participants were asked to answer a self-assessment form, “The Hip Sports Activity Scale” (HSAS), which was used to identify the activity level and the primary sport [27]. The original English version of the HSAS was used. Some participants in this study performed sports such as cross fit, military fitness, climbing and futsal. As these sports are not represented in the HSAS, they were considered as level 4 sports since they are primarily exercised indoors and level 4 is in the middle of the HSAS.

An ethical application for this project was approved by the Central Ethical Review Board at University of Gothenburg (Sweden), case number 071-12, and all participants provided oral and written informed consent.

### Statistical methods

The results are presented as means with standard deviations (SD), except HSAS scores (ordinal), where median and interquartile ranges are presented. Differences in performance between the patients’ arthroscopically treated hip with the non-treated hip for the test of hip strength were analysed using a paired samples *t* test. A paired samples *t* test was also used to detect significant differences for the test of hip strength between the right and the left hip within the matched control group. Differences in performance for the test of hip strength between the patients’ arthroscopically treated hip and the non-treated hip, respectively, with the average value of the right and the left hip in the matched control group were analysed with an unpaired *t* test. Upon analysing the median values regarding HSAS between the groups, Mann–Whitney *U* test was used. A computer program was used for all statistical calculations (SPSS version 21, IBM, USA). The significance level was set to *p* < 0.05. SD values were based on values gathered in the study by Augustsson [2]. Based on a hypothesised 15% difference in hip muscle strength between the involved and non-involved hip, 22 was the estimated number of patients required to achieve a power of 0.90.

## Results

Between June 2016 and September 2017, 44 individuals were enrolled in the study, divided into an observational group of patients (*n* = 22) and a matched control group of healthy individuals (*n* = 22). Descriptive characteristics are presented in Table 1. The participants were active in different sports on various levels, from a recreational to a professional level (3–8 on HSAS). The participants were engaged in soccer, martial arts, running, weight lifting/gym exercises, horseback riding, motorcar racing, crossfit, military fitness, downhill skiing, cross country skiing, handball, futsal, climbing, mountain biking, floorball, tennis, swimrun and beach volley. Some individuals participated in multiple sports.

**Table 1:** Descriptive characteristics of the participants (*n* = 44)

	Observational group (*n* = 22)	Matched control group (*n* = 22)
Males/females	12/10	12/10
Age (years)	33 (± 10)	33 (± 11)
Height (cm)	176 (± 7)	171 (± 10)
Weight (kg)	74 (± 11)	70 (± 12)
HSAS	4 (4)	4.5 (2.25)

Values are expressed as mean ± SD, except for HSAS (ordinal), where median and interquartile range are given

HSAS The Hip Sports Activity Scale

Dynamic hip external rotation strength was significantly lower in the arthroscopically treated hip compared with the healthy side (11.5 versus 12.2 kg, *p* = 0.004) (Table 2). Of the 22 patients in the observational group, 14 (64%) were weaker on the operated side, four (18%) were equally strong, and four (18%) were stronger on the operated side.

**Table 2:** Comparison of mean ± SD external rotation strength between the hip treated with arthroscopy and non-treated hip in the observational group (*n* = 22)

Hip treated with arthroscopy (kg)	Non-treated hip (kg)	Mean diff (kg/%)	95% CI	*p* value
11.5 (± 3.5)	12.2 (± 3.4)	− 0.7/6	− 1.09; − 0.23	0.004

*SD* standard deviation, *Mean diff* mean difference, *CI* confidence interval

For the matched control group, no significant difference was noted in dynamic hip external rotation strength between the right side and the left side (10.8 versus 10.6 kg) (Table 3). Of the 22 participants in the control group, 13 (59%) were stronger on the right side, 7 (32%) were stronger on the left side, and 2 (9%) were equally strong on both sides.

**Table 3:** Comparison of mean ± SD external rotation strength between the right and left hip in the matched control group (*n* = 22)

Right side (kg)	Left side (kg)	Mean diff (kg/%)	95% CI	*p* value
10.8 (± 2.1)	10.6 (± 2)	0.2/2	− 0.16; 0.60	n.s

*SD* standard deviation, *Mean diff* mean difference, *CI* confidence interval, *n.s* not significant

No significant differences in dynamic hip external rotation strength were found between the observational group and the matched control group. This applied when hip muscle strength for both the affected and the unaffected hip in the observational group was compared with the matched control group (Tables 4, 5).

**Table 4:** Comparison of mean ± SD external rotation strength between the arthroscopically treated hip in the observational group versus the average of both hips of the matched control group (*n* = 44)

Observational group (kg)	Matched control group (kg)	Mean diff (kg/%)	95% CI	*p* value
11.5 (± 3.5)	10.7 (± 2)	0.8/7	− 0.94; 2.53	n.s

*SD* standard deviation, *CI* confidence interval, *n.s* not significant

**Table 5:** Comparison of mean ± SD external rotation strength between the non-treated hip in the observational group versus the average of both hips of the matched control group (*n* = 44)

Observational group (kg)	Matched control group (kg)	Mean diff (kg/%)	95% CI	*p* value
12.2 (± 3.4)	10.7 (± 2)	1.44/12	− 0.26; 3.15	n.s

*SD* standard deviation, *CI* confidence interval, *n.s* not significant

There was no significant difference between the two groups regarding the reported HSAS score. The median score of the observational group and matched control group was 4 and 4.5, respectively.

## Discussion

The most important finding of the present study was that full recovery of hip muscle strength is not achieved at return to sports in patients with femoroacetabular impingement surgery. To the best of our knowledge, this is the first study investigating hip muscle strength at return to sports in patients following FAI arthroscopy surgery, compared to the non-treated hip as well as with a matched control group. The results are of clinical relevance, as they underline the importance of post-operative strength training prior to returning to sports in patients with FAI impingement surgery. A study of Domb et al. [11] showed that after hip arthroscopy, a structured step-based rehabilitation program may contribute to good patient-reported hip outcome scores, and a return of patients to their individual sports. Neither functional performance (e.g. different single hop tests) nor hip muscle strength, however, was assessed. A study by Bennell et al. [4] showed a significant difference in outcomes after 14 weeks comparing rehabilitation led by a physiotherapist to self-training after surgical operation of FAI syndrome. However, after 24 weeks, no significant between-group differences were noted. Bennell et al. [4] only used self-assessment tools and not muscle- or strength tests, therefore, eventual benefits of the physiotherapist-prescribed rehabilitation programme on hip strength are not clear. In a clinical commentary, Kuhns et al. [18] recently presented a protocol based on prior studies and expert commentary for rehabilitation after arthroscopic treatment of FAI syndrome. The protocol consisted of a four-phase physical therapy program following a step-wise approach regarding exercises and progression during the rehabilitation process. The proposed protocol, however, remains to be evaluated on patients with FAI syndrome. A randomized controlled trial by Grant et al. [13] investigated an intervention group where the participants were given an 8-week-pre-surgical-training programme before undergoing arthroscopic treatment for FAI syndrome, while the control group were not given any pre-surgical training. Post-arthroscopically all participants in both groups followed the same rehabilitation programme. The intervention group showed a significantly improved isometric knee extension and hip flexion strength compared to the control group. Mansell et al. [22] investigated the effect of arthroscopic surgery versus physiotherapy for patients with FAI syndrome. After 2 years there were no significant differences between the groups, however, patient-reported outcomes of pain, disability, and the perception of improvement rather than functional performance or muscle strength tests were collected. Taken together, there is a lack of studies in the literature that evaluate muscle strength in patients after arthroscopic treatment for FAI syndrome. Accordingly, in a recent review on hip muscle strength in patients with FAI a total of 29 articles were assessed, of which only three related to strength [24]. This is problematic because if hip muscle strength is not tested or documented, it is impossible to answer research questions such as the effect of strength training during FAI rehabilitation. It is plausible there is a strength deficit in muscle groups of the hip other than external rotation as shown in the present study. It is also possible that a strength deficit of the hip muscles exists before the arthroscopic treatment based on prior studies that have shown reduced functionality in patients with FAI syndrome [8, 16, 17, 19, 24]. This in turn raises the question of whether the strength deficit is a result of FAI syndrome or, on the contrary, the FAI syndrome is a cause of insufficient hip muscle strength?

In the present study, no significant differences were noted neither when hip muscle strength for the affected nor the unaffected hip in the observational group were compared with the matched control group. Throughout the literature there is, to our knowledge, only one other study in which hip muscle strength in patients with FAI was compared with normal controls [7]. It was noted that patients with FAI had significantly lower strength than controls for all hip muscle groups, except for internal rotators and extensors. A possible explanation for these contradictory findings is that the in the present study, patients had undergone arthroscopic treatment whereas in the study by Casartelli et al. [7], the patients’ FAI had remained untreated. To establish more definitely hip strength in patients with FAI versus normal controls, future work need to be done comparing strength in a surgery group, a non-operative intervention (physiotherapy) group and a control group.

In the present study, the patients with FAI surgery had reduced hip muscle strength. The results observed is of importance, as these individuals were back in full sports activity with a hip strength deficit that potentially could lead to reinjury or pathologies such as patellofemoral pain [1], osteoarthritis [1, 16] and ACL injury [1, 15]. In this study there were a 6% difference between the arthroscopically treated and non-treated hip. This raises the question of how large a difference can be considered pathological? [28]. It was observed by Almeida et al. [1] that the subjects had a hip external rotation muscle strength deficit of between 5–36%. Nepple et al. [28] considered 10% to be a pathological difference in hip strength. When it comes to muscle strength criteria given for return to sports after ACL reconstruction, 100% strength (of the non-injured leg) on knee extensors as well as knee flexors have been suggested [34]. There exists, however, to our knowledge no muscle strength criteria in the literature describing an acceptable level of hip strength before return to sports following FAI surgery.

A limitation of the present study concerns whether it was the dominant or non-dominant hip that was treated arthroscopically. During the analysis, no significant difference in strength was noted between the non-operated hip of the observational group and the average of both hips of the matched control group. It is conceivable, however, that if the non-dominant hip was the arthroscopically treated, differences would be more significant than if the dominant hip was treated. Studies on hip strength in injury-free athletes have shown, however, only marginal difference between the dominant and the non-dominant side (3–4%) when it comes to isometric hip adduction and abduction strength [35]. Further, since not all sports are represented in the HSAS form, a subjective assessment was made for the missing sports. However, HSAS was chosen because the participants of the observational group used the same tool prior to their arthroscopy and HSAS has been tested in terms of reliability and validity [27]. Another limitation involves the reliability of the measurements by the rubber bands, as they could get worn out and lose their strength over time. There was also a risk for the rubber bands to vary in quality even if the manufacturer was the same throughout all testing sessions. However, the rubber band was calibrated with each test and replaced regularly as a precaution and, further, the Augustsson Strength Test has shown high reliability [2]. In addition, in the study by Augustsson [2], the question of the validity of the Augustsson Strength Test was addressed. It was noted that although a criterion validity test comparing the new device with “gold standard” would have been desirable, no isotonic “gold standard” test of hip strength exists today in the clinical setting. However, in a recent study that assessed different hip exercises, Selkowitz et al. [33] noted that muscle activation (especially the gluteus maximus) was among the highest during the so called “Clam” exercise, which is performed the same way as the Augustsson Strength Test. This in turn validates the Augustsson Strength Test, in that it actually measures the strength of the hip muscles.

The results of this study provide clinical relevance, as they underline the importance of post-operative strength training prior to returning to sports in patients with femoroacetabular impingement surgery. Future studies on FAI rehabilitation specifically designed on interventions aimed at strengthening the hip muscles are needed. Further, it is important to determine the most suitable methods to measure hip muscle strength, what hip muscle groups to measure and what hip muscle strength criteria to use before return to sports after FAI surgery.

## Conclusion

This cross-sectional study shows that at return to sports, patients who have undergone unilateral arthroscopic treatment for FAI syndrome have reduced hip muscle strength. Rehabilitation protocols should, therefore, emphasise post-operative hip muscle strength training. Additional research is needed to determine the consequences of reduced hip strength for the long-term outcome after arthroscopically treated FAI.
